# *Toxoplasma gondii* in Wild Red Squirrels, the Netherlands, 2014

**DOI:** 10.3201/eid2112.141711

**Published:** 2015-12

**Authors:** Marja Kik, Jooske IJzer, Marieke Opsteegh, Margriet Montizaan, Vilmar Dijkstra, Jolianne Rijks, Andrea Gröne

**Affiliations:** Utrecht University, Utrecht, the Netherlands (M. Kik, J. IJzer, M. Montizaan, J. Rijks, A. Gröne);; Dutch National Institute for Public Health and the Environment, Bilthoven, the Netherlands (M. Opsteegh); Dutch Mammal Society, Nijmegen, the Netherlands (V. Dijkstra)

**Keywords:** Toxoplasma gondii, red squirrels, the Netherlands, toxoplasmosis, Sciurus vulgaris, zoonoses, protozoa, parasites

**To the Editor**: *Toxoplasma gondii*, a zoonotic protozoan parasite for which felids are the only definitive hosts, can infect humans and other warm-blooded animals. Transmission usually occurs orally from oocysts shed by felids in water and on food, through tissue cysts in undercooked meat, or transplacentally. In particular, young cats shed oocysts that can sporulate and become infectious within a day, depending on temperature and humidity. Sporulated oocysts can survive in moist soil for months to years (*1*).

In September 2014, the number of dead squirrels reported to the Dutch Wildlife Health Centre and the Dutch Mammal Society increased suddenly. The red squirrel (*Sciurus vulgaris*) is the only species of squirrel endemic to the Netherlands. Members of the public claimed that squirrels were “dropping dead from trees.” Subsequently, the public was encouraged to report and submit dead squirrels. A total of 187 animals were reported through October 2014, of which 37 were submitted for necropsy. Necropsy included macroscopic examination; cytologic analysis of liver, spleen, lungs, and intestinal contents stained with hemacolor (Merck, Darmstadt, Germany); and histologic examination of samples of various organs fixed in formalin, embedded in paraffin, cut into 4-μm sections, and stained with hematoxylin and eosin.

For 8 adult animals, body condition (based on degree of fat storage and muscle development) was good; 12 juveniles were in poor condition. Typically, the trachea contained foam, and lungs were hyperemic and edematous. The liver was enlarged and pale, and the spleen was enlarged. In 13 animals, numerous small crescent-shaped organisms, with eccentrically placed nuclei consistent with tachyzoites of *T. gondii*, were identified by cytology in lung, liver, and spleen (*2*). Main histopathologic findings were pulmonary interstitial lymphoplasmocytic and neutrophilic infiltrates with edema and numerous intra-alveolar macrophages (17/20) and multifocal lymphoplasmocytic infiltrates with necrosis in the liver (13/20). Extensive splenic necrosis was occasionally observed (4/20). Intestines contained mild plasmacytic infiltrates. Numerous tachyzoites consistent with *T. gondii* were present in alveolar macrophages and epithelial cells, splenic macrophages, and hepatocytes. Duplicate slides were stained immunohistochemically by using polyclonal antibodies against *T. gondii* following a standard ABC protocol (*3*). Organisms stained for *T. gondii* in liver, spleen, lungs, and intestine. *Toxoplasma* was not detected in any brain. DNA was isolated (DNeasy Blood and Tissue Kit; QIAGEN, Hilden, Germany) from tissues of 14 squirrels and tested by quantitative PCR (*1*); *T. gondii* DNA was detected in 13. We successfully sequenced the *T. gondii* GRA6 gene for 11 squirrels and identified sequences to clonal type II *T. gondii* previously identified in sheep from the Netherlands (GenBank accession no. GU325790) (*4*). Incidental findings in the animals tested were encephalitis (2/20), coccidiosis (5/20), trauma (6/20), myocarditis (4/20), nephritis (1/20), lymphadenitis (1/20), and intestinal (3/20) and external (5/20) parasites.

The remaining 17 animals showed >1 of the following pathologic conditions: hemorrhages consistent with trauma (12/17), mild to severe intestinal coccidiosis (12/17), pneumonia (3/17), splenitis (1/17), *Taenia martis* cysticerci (1/17), and external parasites (8/17). Immunohistochemistry results for all 17 were negative for *T. gondii*.

On the basis of necropsy and molecular findings, we conclude that 20 of 37 examined squirrels died of disseminated *T. gondii* type II infection. These animals included adults and juveniles and were not restricted to specific geographic areas (Figure). The remaining animals died of trauma (12/17) or other causes (5/17).

**Figure:**
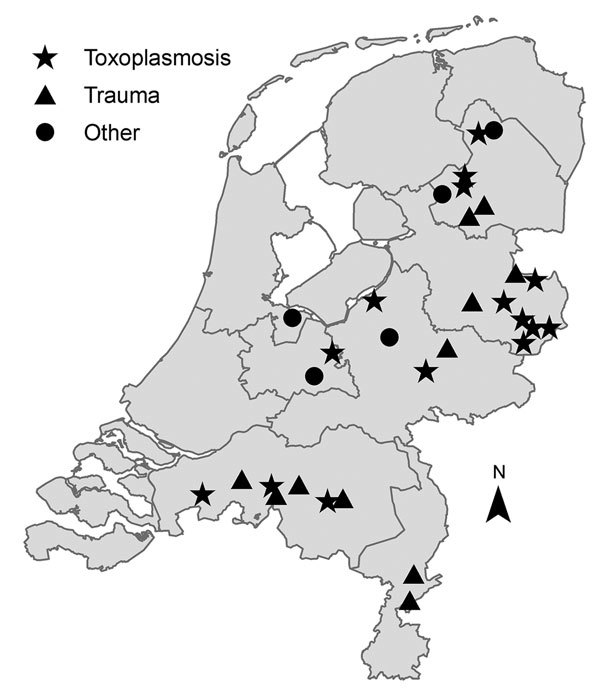
Spatial distribution of wild red squirrels (*Sciurus vulgaris*) investigated for *Toxoplasma gondii* and classified by cause of death, the Netherlands, 2014.

Red squirrels are susceptible to *T. gondii*, and infection can lead to death. However, in our sample, the proportion of squirrels that died of toxoplasmosis (>50%) was higher than in other studies (≈16%) (*5**–**7*). The apparent increase in squirrel deaths and unexpectedly high proportion of fatal *T. gondii* infections suggests a toxoplasmosis outbreak among red squirrels. Possible explanations for this surge in cases include increased exposure to the parasite, increased susceptibility to infection, or increased virulence of the pathogen. Clonal *T. gondii* type II, the strain most frequently involved in human cases and endemic to Europe and North America, was identified. An increased virulence of the pathogen could not be proven (*8*). On the basis of lymphoid hyperplasia in the spleen and lymph nodes, affected squirrels had no signs of immunosuppression. Thus, the most likely explanation is increased exposure to the parasite.

Sources of infection for red squirrels are not known; however, oocysts shed in cat feces may contaminate the nuts, fungi, shoots, and berries that constitute the diet of the squirrel. Stray, unspayed cats are common in the Dutch countryside. More than 3 million domestic cats (*Felis domesticus*) exist in the Netherlands, including several tens of thousands of free-roaming cats that reproduce (*9*). Determining the exact source of infection is important because humans also harvest wild fruits, nuts, and fungi from these areas. This outbreak highlights that contamination of the environment with *T. gondii* oocysts is of concern not only from a public health viewpoint but from a biodiversity perspective as well (*1*,*10*).

## References

[1] Elmore SA, Jones JL, Conrad PA, Patton S, Lindsay DS, Dubey JP. *Toxoplasma gondii*: epidemiology, feline clinical aspects, and prevention. Trends Parasitol. 2010;26:190–6. 10.1016/j.pt.2010.01.009 20202907

[2] Gardiner CH, Fayer R, Dubey JP. Apicomplexa. In: An atlas of protozoan parasites in animal tissues. Washington: American Registry of Pathology; 1988. p. 31–64.

[3] Key M. Immunohistochemical staining methods. In: Kumar GL, Rudbeck L, editors. Immunohistochemical staining methods, 5th ed. Carpinteria (CA): Dako Corporation; 2009. p. 57–60.

[4] Opsteegh M, Langelaar M, Sprong H, den Hartog L, De Craeye S, Bokken G, Direct detection and genotyping of *Toxoplasma gondii* in meat samples using magnetic capture and PCR. Int J Food Microbiol. 2010;139:193–201. 10.1016/j.ijfoodmicro.2010.02.027 20350771

[5] Duff JP, Higgins RJ, Sainsbury AW, Macgregor SK. Zoonotic infections in red squirrels. Vet Rec. 2001;148:123–4 . 11232934

[6] Jokelainen P, Nylund M. Acute fatal toxoplasmosis in three Eurasian red squirrels (*Sciurus vulgaris*) caused by genotype II of *Toxoplasma gondii.* J Wildl Dis. 2012;48:454–7. 10.7589/0090-3558-48.2.454 22493121

[7] Simpson VR, Hargreaves J, Butler HM, Davison NJ, Everest DJ. Causes of mortality and pathological lesions observed post-mortem in red squirrels (*Sciurus vulgaris*) in Great Britain. BMC Vet Res. 2013;9:229. 10.1186/1746-6148-9-229 24238087 PMC4225685

[8] Shwab EK, Zhu XQ, Majumdar D, Pena HF, Gennari SM, Dubey JP, Geographical patterns of *Toxoplasma gondii* genetic diversity revealed by multilocus PCR-RFLP genotyping. Parasitology. 2014;141:453–61. 10.1017/S0031182013001844 24477076

[9] Wildlife Management Unit. Feral and stray cats [in Dutch] [cited 2014 Oct 10]. http://www.faunabeheereenheid.nl/gelderland/Diersoorten/Verwilderde%20kat%20%20en%20zwerfkat%20def.doc/

[10] Carme B, Bissuel F, Ajzenberg D, Bouyne R, Aznar C, Demar M, Severe acquired toxoplasmosis in immunocompetent adult patients in French Guiana. J Clin Microbiol. 2002;40:4037–44 . 10.1128/JCM.40.11.4037-4044.2002 12409371 PMC139686

